# A comparative analysis of hypofractionated versus conventional radiotherapy for cervical cancer in a resource-limited setting: a prospective study

**DOI:** 10.3389/fonc.2025.1552346

**Published:** 2025-06-04

**Authors:** Abba Mallum, Maureen Bilinga Tendwa, Rakiya Saidu, William Swanson, Paul Phan, Heng Li, Twalib Ngoma, Stephen Avery, M. Saiful Huq, John M. Akudugu, Wilfred Ngwa, Luca Incrocci, Mariza Vorster

**Affiliations:** ^1^ Department of Radiotherapy and Oncology, College of Health Sciences, University of KwaZulu Natal, Durban, South Africa; ^2^ Department of Radiotherapy and Oncology, Inkosi Albert Luthuli Central Hospital, Durban, South Africa; ^3^ Faculty of Medicine and Health Sciences, Walter Susilu University, Mthatha, South Africa; ^4^ Department of Complementary Medicine, South Africa Health Product Regulatory Authority (SAHPRA), Pretoria, South Africa; ^5^ Department Obstetrics and Gynaecology, Faculty of Health Sciences, University of Cape Town, Cape Town, South Africa; ^6^ Department of Radiation Oncology, Emory University, Atlanta, GA, United States; ^7^ Department of Radiation Oncology and Molecular Radiation Sciences, Johns Hopkins University, Baltimore, MD, United States; ^8^ Department of Clinical Oncology, Muhimbili University of Health and Allied Sciences, Dar es Salaam, Tanzania; ^9^ Department of Radiation Oncology, University of Pennsylvania, Philadelphia, PA, United States; ^10^ Department of Radiation Oncology, University of Pittsburgh School of Medicine and University of Pittsburgh Medical Center (UPMC) Hillman Cancer Center, Pittsburgh, PA, United States; ^11^ Division of Radiobiology, Department of Medical Imaging and Clinical Oncology, Faculty of Medicine and Health Sciences, Stellenbosch University, Tygerberg, South Africa; ^12^ Brigham and Women’s Hospital Dana-Farmer Cancer Institute, Harvard Medical School, Boston, MA, United States; ^13^ Department of Radiotherapy, Erasmus MC Cancer Institute, Rotterdam, Netherlands; ^14^ Department of Nuclear Medicine, College of Health Sciences, University of KwaZulu Natal, Durban, South Africa

**Keywords:** cervical cancer, conventional radiotherapy, hypofractionated radiotherapy, adverse reactions, overall survival

## Abstract

**Introduction:**

Hypofractionation has potential benefits for cancer patients in low-income countries by reducing treatment duration and resource demands. However, few studies have examined the potential for higher toxicity due to the increased radiation dose per session, particularly in patients with existing health burdens like HIV. This study aimed to evaluate and compare the toxicity profiles of conventionally fractionated radiotherapy (CFRT) and hypofractionated radiotherapy (HFRT) in cervical cancer patients in a low-income setting, facilitating a better understanding of the associated risks and benefits to ensure safe and effective treatment options.

**Methods:**

A prospective cohort study was conducted at Inkosi Albert Luthuli Central Hospital (IALCH) in South Africa from March 2022 to March 2023. A total of 107 patients with confirmed cervical cancer were recruited and randomly assigned to either CFRT (n = 54; 50.50 Gy in 25 fractions) with weekly chemotherapy or HFRT (n = 53; 42.72 Gy in 16 fractions). Additionally, both groups received high-dose-rate (HDR) intracavitary brachytherapy, with doses of 18.00-, 21.00-, or 10.00-Gy boost. Clinical data and adverse events were recorded and analyzed, with statistical significance set at p < 0.05.

**Results:**

The median age at diagnosis was 36.4 (28.2–62.9) years, with 85.0% of patients under 40 years and 86.0% HIV-positive. Most patients in both groups presented with stage IIB and grade II disease. HFRT patients completed radiotherapy significantly faster (median, 35 days) than CFRT patients (median, 62 days) (p < 0.001). Both groups experienced similar rates of gastrointestinal (GI), genitourinary (GU), and skin toxicity, although significant differences were found in GI (p = 0.005) and GU (p = 0.01) side effects. Vaginal stenosis was more common in the CFRT group (51.9%) than in the HFRT group (43.4%). Both groups showed comparable clinical responses, recurrence-free survival, and absence of residual disease within 12 months.

**Conclusion:**

HFRT (42.72 Gy in 16 fractions) offers comparable outcomes to CFRT (50.50 Gy in 25 fractions) with a shorter treatment duration, making it a feasible option in resource-limited settings.

## Introduction

Cervical cancer, primarily driven by human papillomavirus (HPV), remains a significant public health threat in Sub-Saharan Africa (1–3). Limited access to healthcare and resource constraints hinder early diagnosis and treatment, contributing to high mortality rates (1). While advancements in treatment options have been made, the region continues to face challenges in addressing this issue (1, 3). Hypofractionated radiotherapy (HFRT) offers potential benefits for cancer patients in low-income countries by reducing treatment duration and resource demands (3, 4). While HFRT has shown a toxicity profile comparable to that of conventionally fractionated radiotherapy (CFRT) (5, 6), there is limited research on the potential for increased toxicity, particularly in African populations with pre-existing health conditions such as HIV (7, 8). Therefore, understanding the toxicity profile of HFRT is essential to balance the benefits of shorter treatment times with the risks, ensuring safe and effective treatment options in these regions, where cervical cancer disproportionately impacts the population. According to GLOBOCAN 2022 data, Sub-Saharan Africa accounted for over 118,013 new cervical cancer cases and approximately 76,189 deaths (9), representing 35 new cases per 100,000 women and 23 deaths per 100,000 women annually (10). It is the fourth most common cancer among women worldwide, with an estimated 604,000 new cases and 342,000 deaths in 2022 (9). With the global burden of cervical cancer projected to increase substantially by 2030, there is an urgent need for effective and accessible treatment strategies (3, 11, 12). HFRT emerges as a promising alternative, offering potential advantages in terms of reduced treatment duration and cost and improved patient adherence (13). Further research is imperative to evaluate its effectiveness and feasibility in managing cervical cancer within the African context.

Cervical cancer management often necessitates a multimodality approach integrating surgery, chemotherapy, and radiotherapy (14). Combining these treatment modalities has been shown to improve overall survival and local control compared to radiotherapy alone (15, 16). The choice of surgical intervention depends on the tumor’s stage and size (16, 17). For instance, early-stage malignancies may be suitable for radical trachelectomy, which prioritizes curative intent while preserving fertility (16, 18). Alternatively, depending on the severity of the disease, a simple or radical hysterectomy, with or without the removal of surrounding tissues, may be performed. For locally advanced cervical cancer, concurrent chemoradiation therapy is the established standard of care (16).

Radiotherapy plays a pivotal role in cervical cancer treatment, offering adaptability in its application. It can be employed curatively to eradicate cancer cells, reduce tumor size, and minimize recurrence risk (16, 19). External beam radiotherapy (EBRT) and brachytherapy, including high-dose-rate (HDR) brachytherapy, are key techniques employed in cervical cancer treatment, each with its own advantages (16, 19–21).

EBRT delivers high-energy radiation beams from an external source, precisely targeting the tumor and surrounding tissues at risk (pelvic region) (19). This technique allows for the delivery of an intensity-modulated dose of radiation to the target volume while minimizing exposure to healthy tissues (19). In contrast, brachytherapy involves the placement of radioactive implants directly within the tumor site. This targeted approach enables the delivery of a concentrated dose of radiation to cancer cells, further enhancing the therapeutic ratio (20).

The combination of EBRT and brachytherapy, particularly with HDR brachytherapy’s ability to deliver a concentrated dose in fewer sessions, offers an improved strategy for better outcomes in cervical cancer patients (19, 20). These radiotherapy techniques can be utilized within either CFRT or HFRT regimens, depending on specific treatment goals and patient characteristics (21).

While CFRT with weekly platinum-based chemotherapy remains the standard of care for cervical cancer, emerging evidence supports the efficacy of HFRT in both breast and prostate cancers (5, 13, 21–28). For breast cancer, recent phase III trials, including the NCT00793962 study, have demonstrated that postmastectomy HFRT is non-inferior to CFRT in high-risk patients, providing similar clinical outcomes while offering a more cost-effective treatment option (23). This trend is further supported by meta-analyses indicating that HFRT not only maintains efficacy but also reduces the incidence of side effects such as breast edema (24). In prostate cancer, studies have shown that HFRT regimens yield comparable biochemical control and toxicity profiles to traditional approaches, reinforcing its role as a viable treatment alternative (5, 26–28). Furthermore, HFRT has been shown to deliver outcomes comparable to those of CFRT while potentially reducing treatment duration and associated healthcare costs (13). Kavuma et al. (2021) found no statistical difference in toxicity profiles between CFRT and HFRT for the treatment of locally advanced cervical cancer (6). Moreover, HFRT was associated with a shorter overall treatment duration and reduced gastrointestinal side effects, including diarrhea, abdominal pain, and fecal incontinence, enhancing patient convenience. In a phase 2 trial, HFRT with concurrent chemotherapy demonstrated a low rate of acute grade III or higher toxic effects, significantly lower than CFRT (29).

Consequently, HFRT represents a promising advancement in radiotherapy for cervical cancer, offering effective treatment with a manageable toxicity profile. However, there is a notable scarcity of studies on HFRT for cervical cancer in African countries. Therefore, this study aimed to evaluate the treatment outcomes and toxicity profiles of cervical cancer patients treated with either CFRT (50.50 Gy in 25 fractions) or HFRT (42.72 Gy in 16 fractions) at Inkosi Albert Luthuli Central Hospital (IALCH) in Durban, South Africa. Understanding the toxicity profile of HFRT is essential to balance the benefits of shorter treatment times with the potential risks, ensuring safe and effective treatment options for patients in resource-limited regions. The findings from this study could serve as a valuable model for the broader adoption of HFRT across Africa, improving access to cervical cancer treatment and enhancing patient care outcomes.

## Materials

### Study design

This prospective randomized cohort study was conducted at IALCH in Durban, South Africa, from March 2022 to March 2023.

### Ethical considerations

The study was approved by the Institutional Review Board of the University of KwaZulu Natal (UKZN), and all patients provided written informed consent prior to enrolment. The study adhered to the principles of the Declaration of Helsinki regarding the ethical conduct of research involving human subjects.

### Patient population

A total of 107 patients with histologically confirmed cervical cancer, according to the International Federation of Gynaecology and Obstetrics (FIGO) staging system (30), were enrolled for treatment at IALCH. Patients were recruited through a multidisciplinary team (MDT) and randomly assigned to receive either CFRT and weekly chemotherapy (standard treatment) or HFRT using block randomization. Patients were enrolled in an alternating sequence between the two treatment arms to ensure an equal distribution. As a result, the CFRT arm included a total of 54 patients, while the HFRT arm had 53 patients.

### Patient selection

Inclusion criteria included women 18 years and above with histologically confirmed locally advanced cervical cancer (staged IB3-IVA) and Eastern Cooperative Oncology Group (ECOG) performance status (PS) 0–2. All participants were required to have no distant metastasis as determined by CT scans or fluorodeoxyglucose positron emission tomography (FDG-PET/CT) scans. Additionally, eligible participants must have provided informed consent and committed to attending follow-up visits at 3, 6, 12, and 60 months post-treatment. Exclusion criteria included radiological evidence of distant metastasis, a history of inflammatory bowel disease, neuro-endocrine histology, weight > 145 kg, previous pelvic radiotherapy, bilateral hip prostheses, or unwillingness to participate in follow-up.

### Treatment protocols

Patients were randomly assigned to one of two treatment arms: HFRT (n = 53; 42.72 Gy in 16 fractions) (31) or CFRT (n = 54; 50.50 Gy in 25 fractions). Both groups underwent EBRT using the volumetric modulated arc therapy (VMAT) technique. In addition, the CFRT group received weekly concurrent cisplatin (40 mg/m^2^). However, to reduce the risk of exacerbated toxicity from combining higher fractional doses in HFRT with concurrent chemotherapy and to avoid uncertainties in cisplatin dose adjustments, the HFRT group did not receive weekly cisplatin.

To maximize tumor control while minimizing radiation exposure to surrounding tissues, both groups received HDR intracavitary brachytherapy with either 18.00 Gy in two fractions or 21.00 Gy in three fractions, depending on tumor size, response to EBRT, and overall treatment tolerance. Additionally, patients with complete cervical os destruction due to the tumor or severe vaginal stenosis that could be dilated were administered a targeted boost dose of 10.00 Gy in five fractions of EBRT as part of the treatment protocol.

### Treatment planning and delivery

The treatment planning of VMAT was conducted using the Eclipse treatment planning system (Varian Medical Systems, Palo Alto, CA, USA), and the radiation treatment was delivered by a dynamic multileaf collimator with photon beam energy of 6 MV. For HDR brachytherapy, the Varian GammaMedplus™ iX system (Palo Alto, CA, USA) was used.

Patient positioning and immobilization included the use of a knee rest with ankle support and a headrest, ensuring a reproducible setup. Bladder filling (250 mL of water) was scheduled before CT simulation and daily treatment to minimize bladder volume variations.

The gross tumor volume (GTV) included the cervix, the uterus, and any gross nodal disease, with pelvic lymph nodes defined as the GTV-N. The clinical target volume (CTV)/planned target volume (PTV) included a 0.7- to 1.0-cm radial margin around the GTV, the upper half of the vagina, the parametrium, and regional lymph nodes.

For treatment field definition, the standard field extended from L4–5 interspace to 3 cm below the most distal site of the disease. In cases of para-aortic lymph node metastasis below the renal hilum, an extended-field radiotherapy plan was applied, extending up to T12-L1 interspace.

The CFRT group received a total dose of 50.50 Gy in 25 fractions over 5 (25 days) weeks, while the HFRT group received 42.72 Gy in 16 fractions over 3.2 (16 days) weeks, both delivered to the PTV.

To minimize the risks of late toxicities associated with the larger fraction doses in HFRT while maximizing dose escalation with minimal tissue damage in CFRT, the CFRT arm received a VMAT simultaneous integrated boost (SIB) to the GTV-T and GTV-N, delivering approximately 50.50 Gy in 25 fractions. In contrast, the HFRT arm did not receive SIB. Dose constraints for organs at risk (OARs) were established for both the CFRT arm and HFRT groups (**Table 1**).

**Table 1:** Dose constraints for CFRT and HFRT (32, 33).

Body Organ	CFRT	HFRT
Bladder	V_65%_ < 50%	D_max_ < 42.8 Gy V_38.5Gy_ < 50% V_34.5Gy_ < 75%
Rectum	V_50%_ < 50%	D_max_ < 42.8 Gy V_38.5 Gy_ < 50% V_34.5Gy_ < 85% V_26.5Gy_ < 95%
Femur heads	D_max_ < 52 Gy	D_max_ < 42.8 Gy
Bowel		D_max_ < 42.8 Gy V_34.5Gy_ < 100cc (maximally V_34.5Gy_ < 250cc)

CFRT, conventionally fractionated radiotherapy; HFRT, hypofractionated radiotherapy.

### Brachytherapy and dose delivery

After completing EBRT (CFRT or HFRT), HDR brachytherapy was delivered using iridium-192. For patients with pre-existing gastrointestinal (GI) and/or genitourinary (GU) comorbidities, as well as those who could undergo multiple brachytherapy insertions without logistical challenges, treatment included a median cumulative dose of 21 Gy in three fractions to point A. In contrast, patients with a smaller tumor burden, a good response post-EBRT, or logistical constraints received 18 Gy in two fractions to point A. For those patients who received 21 Gy in three fractions, a dose constraint was applied, ensuring that the bladder and rectum received 2cc < 7.0 Gy and 2cc < 5.3 Gy, respectively. For those receiving 18 Gy in two fractions, the dose constraints ensured bladder and rectal doses remained 2cc < 9.0 Gy and 2cc < 6.3 Gy per fraction, respectively. The median cumulative dose and biologically equivalent dose (BED) in 2-Gy fractions were 73.4 and 81.1 Gy, respectively, assuming an α/β ratio of 10 Gy for tumor control (32, 33).

### Data collection

Demographic and clinical data were collected at baseline, including age, FIGO stage, histology, and HIV status. Treatment-related data, including radiotherapy fractionation, chemotherapy regimens, and any adverse events, were recorded throughout the treatment period.

### Outcome measures

The primary endpoints were the incidence of grade II or higher GI, GU, and skin reactions, as defined by the National Cancer Institute’s Common Terminology Criteria for Adverse Events, version 5.0 (34). Secondary endpoints included vaginal stenosis, radiation-induced proctitis, response rates, and recurrence-free survival.

### Statistical analysis

Statistical analyses were conducted using R Statistical Computing Software (version 3.6.3). Descriptive statistics, including minimum, maximum, quartiles, interquartile range, mean, standard deviation, and coefficient of variation, were calculated for numerical variables. Categorical variables were summarized using counts and percentage frequencies. To compare groups, appropriate statistical tests were employed: t-tests or Wilcoxon tests for comparing means or medians and chi-square tests or Fisher’s exact tests for comparing categorical variables. All inferential statistical analyses were conducted at a 5% significance level. *Post-hoc* analyses were performed using row-wise paired z-tests when necessary.

## Results

### Sociodemographic characteristics

**Figure 1:**
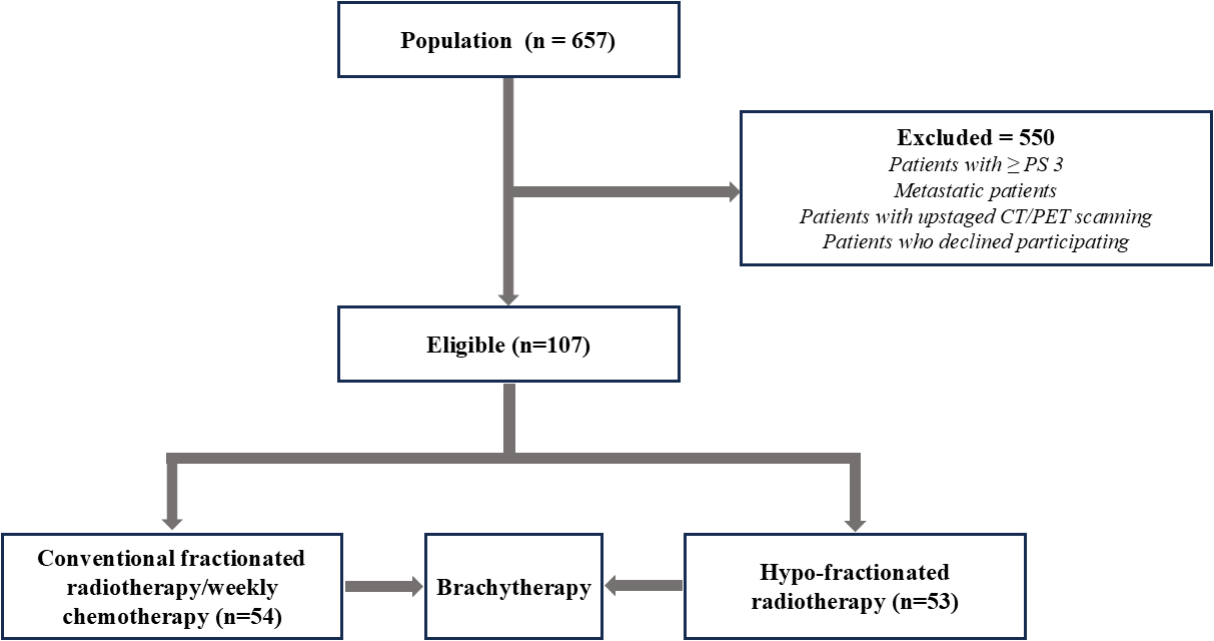
Patient enrolment and eligibility flow diagram.

Between March 2022 and March 2023, 657 patients were registered for cervical cancer treatment at IALCH. Of these, 107 met the inclusion criteria and were randomly assigned to receive either CFRT (n = 54; 50.50 Gy in 25 fractions) or HFRT (31) (n = 53; 42.72 Gy in 16 fractions) (**Figure 1**).

The majority of patients in both groups were younger than 40 years (85.0%), with similar proportions in the CFRT (42.1%) and HFRT (42.9%) groups (**Table 2**). Most patients were of African descent (87.9%), comprising 44.9% in the CFRT group and 43.0% in the HFRT group. The overall median age of patients was 36.4 years (range, 28.2-62.9 years), with the HFRT group having a median age of 35.2 years and the CFRT group having a median age of 32.6 years. The youngest patient (28 years) was in the CFRT group, while the oldest (92.67 years) was in the HFRT group.

**Table 2:** Demographic characteristics of the women included in this study (*n* = 107).

EBRT dose	Overall (n = 107)	CFRT (n = 54)	HFRT (n = 53)	p-Value (<0.05)
Age groups				0.616
≤40 years	91 (85.0%)	45 (42.1%)	46 (42.9%)	
>40 years	16 (15.0%)	9 (8.5%)	7 (6.5%)	
Age in years				
Median (Q1–Q3)	36.4 (28.2–62.9)	32.6 (30.7–60.2)	35.2 (28.7–62.3)	0.006
n (min–max)	107 (28.0–92.6)	54 (28.0–73.0)	53 (34.5–92.6)	
Race				0.385
African	94 (87.9%)	48 (44.9%)	46 (43.0%)	
Indian/Asian	10 (9.3%)	6 (5.6%)	4 (3.7%)	
White	2 (1.9%)	0 (0.0%)	2 (1.9%)	
Colored	1 (0.9%)	0 (0.0%)	1 (0.9%)	
Weight				
Median (Q1–Q3)	80.0 (65.0–101)	78.0 (64.3–97.3)	88.0 (65.0–110)	0.118
n (min–max)	107 (45.0–145)	54 (45.0–135)	53 (45.0–145)	
HIV status				0.152
Negative	15 (14.0%)	5 (4.7%)	10 (9.3%)	
Positive	92 (86.0%)	49 (45.8%)	43 (40.2%)	
Performance status				0.281
0	1 (0.9%)	1 (0.9%)	0 (0.0%)	
1	62 (57.9%)	28 (26.2%)	34 (31.7%)	
2	44 (41.1%)	25 (23.4%)	19 (17.7%)	
Comorbidity				0.003
No	87 (81.3%)	50 (46.7%)	37 (34.6%)	
Yes	20 (18.7%)	4 (3.7%)	16 (15.0%)	

Interquartile range (Q1–Q3), minimum (min), maximum (max), and standard deviation (SD). p-Value represents a comparison between the entire CFRT and HFRT groups across various factors, including age group, age in years, race, weight, HIV status, performance status, and comorbidities.

EBRT, external beam radiotherapy; CFRT, conventionally fractionated radiotherapy; HFRT, hypofractionated radiotherapy.

The weight of patients ranged from 45.0 to 145 kg in the CFRT group and from 45.0 to 135 kg in the HFRT group. A high percentage of patients were HIV-positive (86%) across both groups. Additionally, most patients in both groups had a PS of 1, with 26.2% in the CFRT group and 31.8% in the HFRT group. The percentage of patients presenting with confounding comorbidities differed significantly (0.003) between the groups, with a higher proportion of patients in the HFRT group (15.0%) having comorbidities compared to the CFRT group (3.7%).

The chi-squared test for independence revealed significant differences (p = 0.021) in the distribution of cancer stages among the different treatment groups (**Table 3**). The majority of patients in both groups presented with stage IIB disease (CFRT, 21.5%; HFRT, 20.6%), followed by stage IIIB (CFRT, 10.3%; HFRT, 22.4%). Conversely, only a few patients presented with stage IIA2 (CFRT, 0.9%; HFRT, none) or stage IVA (HFRT, 0.9%). Additionally, most patients were classified as having grade II (moderately differentiated squamous cell carcinoma) (82.2%), with 42.1% in the CFRT group and 40.1% in the HFRT group. Notably, one patient (1.9%) in the HFRT group died 7 months post-treatment due to other causes.

**Table 3:** Patient tumor characteristics and treatment approaches.

EBRT dose	Overall (N = 107)	CFRT (N = 54)	HFRT (N = 53)	p-Value
Survival status				0.495
Alive	106 (99.1%)	54 (50.5%)	52 (48.6%)	
Demised	1 (0.9%)	0 (0.0%)	1 (0.9%)	
Stages				0.021
IB3	3 (2.8%)	3 (2.8%)	0 (0.0%)	
IIA1	13 (12.1%)	9 (8.4%)	4 (3.7%)	
IIA2	1 (0.9%)	1 (0.9%)	0 (0.0%)	
IIB	45 (42.1%)	23 (21.5%)	22 (20.6%)	
IIIA	5 (4.7%)	4 (3.8%)	1 (0.9%)	
IIIB	35 (32.7%)	11 (10.3%)	24 (22.4%)	
IIIC	4 (3.7%)	3 (2.8%)	1 (0.9%)	
IVA	1 (0.9%)	0 (0.0%)	1 (0.9%)	
Grading				0.806
Well-differentiated	7 (6.5%)	4 (3.7%)	3 (2.8%)	
Moderately differentiated	88 (82.2%)	45 (42.1%)	43 (40.1%)	
Poorly differentiated	12 (11.2%)	5 (4.7%)	7 (6.5%)	
Concurrent chemo				
Median (Q1–Q3)	1.00 (0–4.00)	4.00 (3.00–5.00)	0 (0–0)	<0.001
n (min–max)	106 (0–6.00)	53 (2.00–6.00)	53 (0–0)	
Brachytherapy dose				<0.001
18 Gy	35 (32.7%)	1 (0.9%)	34 (31.8%)	
21 Gy	66 (61.7%)	52 (48.6%)	14 (13.1%)	
10.00 Gy (boost EBRT)	6 (5.6%)	1 (0.9%)	5 (4.7%)	
Days of RT completed in days				
Mean ± SD (CV%)		62.5 ± 4.08 (6.5)		
Median (Q1–Q3)	57.0 (35.0–62.0)	62.0 (60.0–65.0)	35.0 (33.0–52.0)	<0.001
n (min–max)	107 (21.0–75.0)	54 (53.0–75.0)	53 (21.0–60.0)	
Time to complete radiation				<0.001
< 56 days	52 (48.6%)	3 (2.8%)	49 (45.8%)	
>56 days	55 (51.4%)	51 (47.7%)	4 (3.7%)	

p-Value represents a comparison between the entire CFRT and HFRT groups across various factors, including survival status, cancer stages, grading, concurrent chemotherapy, brachytherapy dose, days of RT completed in days, and time to complete radiation.

EBRT, external beam radiotherapy; CFRT, conventionally fractionated radiotherapy; HFRT, hypofractionated radiotherapy.

There were significant differences (p < 0.001) in HDR brachytherapy dose regimens between the HFRT and CFRT groups. The majority of patients (61.7%) received the 21-Gy regimen, compared to the 18-Gy regimen (32.7%) and a 10-Gy boost dose (5.6%). A greater percentage of CFRT patients (48.6%) received the 21-Gy radiation dose than HFRT patients (13.1%). Conversely, a higher percentage of HFRT patients (31.8%) received the 18-Gy dose compared to CFRT patients (0.9%). More HFRT patients (4.7%) received a 10-Gy boost dose than CFRT patients (0.9%).

The HFRT group had a statistically significant (p < 0.001) shorter median time to radiotherapy completion (35 days, range, 33.0–52.0 days) compared to the CFRT group (62 days, range, 60.0–65.0 days).

At 6 months post-radiotherapy (**Table 4**), the overall clinical response rate showed that the majority of patients (74.8%) achieved complete clinical response, with lower rates of partial response (23.4%), residual disease (0.9%), and death (0.9%). Both treatment groups exhibited comparable complete response rates of 74.8% (CFRT, 32.9%; HFRT, 35.8%) and partial response rates of 23.4% (CFRT, 11.2%; HFRT, 12.2%). However, one patient (0.9%) in the HFRT group had residual disease, and another succumbed to her condition.

**Table 4:** Patient treatment response and adverse reactions.

EBRT dose	Overall (N = 107)	CFRT (N = 54)	HFRT (N = 53)	p-Value
Post last XRT clinical response rate at 6 months				0.690
Completely clinical response	80 (74.8%)	42 (32.9%)	38 (35.8%)	
Partial clinical response	25 (23.4%)	12 (11.2%)	13 (12.2%)	
Residual disease	1 (0.9%)	0 (0.0%)	1 (0.9%)	
Demised	1 (0.9%)	0 (0.0%)	1 (0.9%)	
Residual disease				0.460
No	82 (76.6%)	43 (40.2%)	39 (36.4%)	
Yes	25 (23.4%)	11 (10.3%)	14 (13.1%)	
Post last RT clinical response rate at 12 months				0.540
Free	86 (80.4%)	45 (42.1%)	41 (38.3%)	
Recurrence	20 (18.7%)	9 (8.4%)	11 (10.3%)	
Died	1 (0.9%)	0 (0.0%)	1 (0.9%)	

At 12 months follow-up, all patients had computer tomography/fluorodeoxyglucose-18 positron emission tomography (CT/FDG-PET scans) scans to rule out distant metastasis. p-Value represents a comparison between the entire CFRT and HFRT groups across various factors, including post last Radiotherapy (XRT) clinical response rate at 6 months, residual disease, and post last RT clinical response rate at 12 months.

CFRT, conventionally fractionated radiotherapy; HFRT, hypofractionated radiotherapy; EBRT, external beam radiotherapy.

At 12 months post-treatment, 23.4% of patients had residual disease (CFRT, 10.3%; HFRT, 13.1%). Meanwhile, 80.4% of patients achieved disease-free survival (CFRT, 42.1%; HFRT, 38.3%), while 18.7% experienced disease recurrence (CFRT, 8.4%; HFRT, 10.3%).

**Figure 2:**
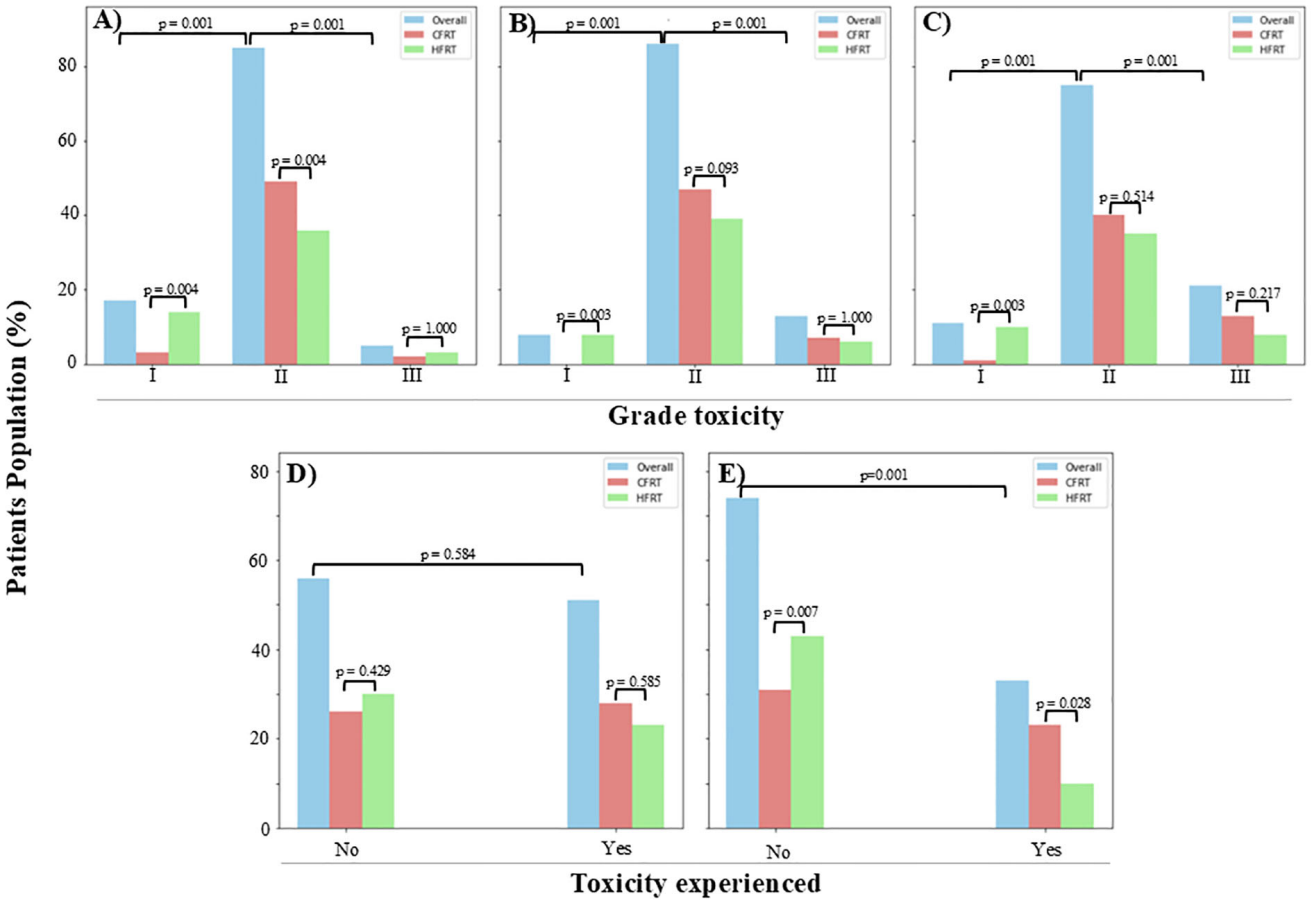
An illustration of toxicity levels at 12 months post-radiotherapy, with the majority of patients exhibiting grade II toxicities. Grade III toxicity levels were comparable between the two groups for **(A)** gastrointestinal (GI) (CFRT, 1.9%; HFRT, 2.8%), **(B)** genitourinary (GU) (CFRT, 6.5%; HFRT, 5.6%), **(C)** skin (CFRT, 12.1%; HFRT, 7.5%), and **(D)** vaginal stenosis (CFRT, 26.2%; HFRT, 21.5%). However, a statistically significant difference (p = 0.028) was observed in **(E)** proctitis rates, with CFRT patients experiencing a higher incidence (21.5%) compared to HFRT patients (9.3%). CFRT, conventionally fractionated radiotherapy; HFRT, hypofractionated radiotherapy.

A statistically significant difference (p = 0.005) was found in the overall incidence of GI, GU (p = 0.01), and skin toxicity (p = 0.01) when comparing all grades (I, II, and III) collectively for both the CFRT and HFRT groups. Across all three toxicity types (GI, GU, and skin), the majority of patients presented with grade II toxicity (p = 0.001) compared to grades I or III (**Figure 2**).

When examining specific GI toxicities, a significant difference was observed between the CFRT and HFRT groups for both grade I (p = 0.004; overall, 15.9%; CFRT, 2.8%; HFRT, 13.1%) and grade II (p < 0.001; overall, 79.4%; CFRT, 45.8%; HFRT, 33.6%) toxicities. However, no significant difference was found in grade III GI toxicities between the two groups (p = 1.0; overall, 4.7%; CFRT, 1.9%; HFRT, 2.8%), and no patients experienced grade IV GI toxicity.

Similarly, GU toxicity showed a statistically significant difference (p = 0.01) between the groups with patients presenting with grade I (7.5%), grade II (80.4%), and grade III (12.1%) adverse effects. The majority of patients had grade II GU toxicity (CFRT, 43.9%; HFRT, 36.5%), followed by grade III (CFRT, 6.5%; HFRT, 5.6%), and a smaller proportion with grade I (HFRT, 7.5%; CFRT, none), while none had grade IV.

A significant difference (p = 0.012) was also noted in skin reaction adverse effects, with patients presenting at grade I (10.3%), grade II (70.1%), and grade III (19.6%). Grade II skin toxicity was the most common (CFRT, 37.4%; HFRT, 32.7%), followed by grade III (CFRT, 12.1%; HFRT, 7.5%), grade I (CFRT, 0.9%; HFRT, 9.4%), and no grade IV in both groups of the study.

Vaginal stenosis, defined as a narrowing of the vaginal length to less than the normal range of 8 to 9 cm, was observed in nearly half of the patients (47.7%), with 26.2% in the CFRT group and 21.5% in the HFRT group.

Overall, 30.8% of patients experienced proctitis. A significantly higher incidence of proctitis was observed in the CFRT group (21.5%) compared to the HFRT group (9.3%, p = 0.02). Of the patients, 69.2% did not develop this condition. Notably, all patients presenting with proctitis symptoms underwent colonoscopy for confirmation.

## Discussion

This prospective cohort study investigated the feasibility of applying HFRT in comparison to CFRT with weekly cisplatin, specifically by investigating the toxicity profile for cervical cancer patients in a low-income country setting, such as IALCH. As expected, most patients were of African descent, reflecting the predominantly African demographic of the study population. The majority of patients in both groups were younger than 40 years, aligning with existing data on cervical cancer prevalence (35, 36). This is likely due to the significant role of HPV infection, which is more prevalent in younger women (37). Notably, 78% of cervical cancer cases in women under 40 occurred between the ages of 30 and 39, with 21% occurring in women aged 20 to 29 (37). Early sexual activity and multiple sexual partners, risk factors for persistent HPV infection, contribute to the increasing incidence of cervical cancer in younger populations (38). Additionally, women living with HIV are six times more likely to develop cervical cancer compared to the general population (39). Given the high prevalence of HIV in the study population, it is unsurprising that most cervical cancer patients were HIV positive.

In addition to presenting with grade II cervical cancer, most patients in the study also presented with advanced stages IIB or III. This finding aligns with previous reports of patients in low- and middle-income countries presenting with progressive disease at diagnosis (40–43). Limited awareness of cervical cancer and its symptoms can contribute to delayed presentation, hindering early detection and treatment (43). As highlighted in our previous work, geographical limitations and inadequate access to radiotherapy services, particularly in rural areas, can further impede timely treatment in Sub-Saharan Africa (44, 45). Implementing strategies to increase access to radiotherapy services, including hypofractionated treatment schedules, could help alleviate these challenges and improve patient outcomes (3, 44, 45). HFRT offers the potential to reduce treatment duration and associated healthcare costs compared to CFRT (3, 4). In this study, HFRT patients completed radiotherapy significantly faster than those receiving CFRT (p < 0.001). The median time to radiotherapy completion was 35 days (range, 33.0–52.0 days) for HFRT and 62 days (range, 60.0–65.0 days) for CFRT. This reduction in treatment time can translate into logistical and operational savings, such as lower patient transportation costs and more efficient use of clinic resources and optimal survival outcome (3, 44, 45).

While CFRT and HFRT have demonstrated comparable treatment outcomes (6, 13), the decision to incorporate brachytherapy as a curative approach for cervical cancer is influenced by factors such as patient characteristics, cancer stage, and tumor size (46, 47). Most patients in this study exhibited favorable performance status (PS < 2) and underwent either CFRT or HFRT, with a higher proportion of comorbidities observed among HFRT patients (15.0%) compared to CFRT patients (3.7%). Significantly more patients in the CFRT (48.6%) group received a total dose of 21 Gy, whereas the HFRT (31.8%) group had a larger proportion receiving 18 Gy. Additionally, a 10-Gy boost dose was more commonly administered to HFRT (4.7%) patients. Despite one patient death in the HFRT group, both groups experienced comparable complete clinical response and partial response rates, supporting earlier findings that HFRT is comparable to CFRT in terms of toxicities and response rate (5, 48, 49).

While both CFRT and HFRT have demonstrated comparable treatment outcomes in cervical cancer, their toxicity profiles may differ (50–53). Emerging evidence suggests that HFRT, particularly when combined with brachytherapy, may offer a more favorable safety profile compared to CFRT (54, 55).

Several studies have reported lower rates of GI and GU toxicity with HFRT regimens (51, 54). For instance, Souhami et al. (2005) conducted a long-term analysis of 282 cervical cancer patients treated with HFRT and brachytherapy (54). At 15 years, the overall GU toxicity rate was 8%, and the GI toxicity rate was 15%. These findings indicate that HFRT can achieve durable local control while maintaining acceptable toxicity levels (54).

More recently, Gandhi et al. (2022) (51) reported promising tolerability in a 50-patient, single-arm prospective study of HFRT for cervical cancer, showing low rates of acute grade II and III GI (20% and 10%) and GU (10% and 6%) toxicities. Late grade II GI and GU toxicities were also manageable at 12% and 6%, respectively (51). While grade I and II toxicities are often deemed less clinically significant than severe events, they can still impact quality of life and necessitate management (56, 57).

Our study revealed a significant difference in grade II toxicity. Of the 79.4% of patients experiencing grade II GI toxicity, the incidence was higher in the CFRT group (45.8%) compared to the HFRT group (33.6%). Similarly, grade II GU toxicity, observed in 80.4% of patients, was more prevalent in the CFRT group (45.8%) than in the HFRT group (33.3%). In contrast, HFRT patients exhibited a higher percentage of grade I GI toxicity (13.1%) than CFRT patients. Compared to Gandhi et al. (2022) (51), our study demonstrated a higher percentage of HFRT patients with grade II GI toxicity (33.6% vs. 20%), although grade III GI toxicity remained low in both studies (2.8% vs. 10%). We also observed a higher incidence of grade II GU toxicity in our HFRT group (36.5%) compared to Gandhi et al., while grade III GU toxicity rates were comparable (5.6% vs. 6%).

It is important to acknowledge that our study included a larger patient population than that of Gandhi et al. (51), and a significant proportion of our patients had comorbidities including HIV (86.0%). These factors likely contributed to the observed differences in toxicity profiles between our study and the findings of Gandhi et al.

In another comparative study involving 43 cervical cancer patients (CFRT, n = 26; HFRT, n = 17), a higher proportion of patients receiving CFRT experienced upper GI (CFRT, 26.9% vs. HFRT, 17.9%), lower GI (CFRT, 34.6% vs. HFRT, 5.9%), GU (CFRT, 19.2% vs. HFRT, 5.9%), and skin reaction toxicities (CFRT, 3.9% vs. HFRT, 0.0%) compared to those receiving HFRT (58). Similar skin reaction percentages were observed in our study, with CFRT at 37.7% and HFRT at 32.7%. Additionally, a higher proportion of CFRT patients experienced vaginal stenosis and proctitis compared to HFRT patients. Although limited studies have examined these specific toxicities, Williamson et al. (2021) reported vaginal stenosis in 38% of patients within the first year of treatment (59). Huang et al. (2016) found that the incidence of radiation proctitis in cervical cancer patients treated with concurrent chemoradiotherapy was significantly influenced by treatment parameters. These findings suggest that HFRT, with its altered fractionation schedule and reduced cumulative radiation dose to the rectum, could potentially minimize the risk of proctitis (60).

The variability in toxicity levels reported across studies comparing HFRT and CFRT in cervical cancer treatment can be attributed to several factors, including patient demographics, sample sizes, treatment durations, and multimodal treatment strategies (61, 62). These variables must be considered when evaluating and comparing the adverse reactions and safety profiles of both treatment regimens.

While radiation therapy can be associated with adverse effects (62), the majority of patients in our study attained comparable complete clinical response, recurrence-free survival at 12 months, and an absence of residual disease in both the CFRT and HFRT groups. These findings support the viability of HFRT (42.72 Gy in 16 fractions) as an effective treatment option, highlighting the need for further clinical investigation and the mitigation of associated challenges to enhance its application in clinical practice.

### Strength and limitations

The study demonstrated a favorable toxicity profile for hypofractionation, with no grade 4 toxicities reported. This suggests that HFRT is a safe and viable alternative to CFRT, even for patients with high weight and comorbidities. The shorter treatment duration also provides significant time and cost efficiency, essential in resource-limited environments, while comparable toxicity profiles, and similar response rates to CFRT reinforce HFRT as an effective shorter-course alternative. Additionally, the study’s inclusion of a diverse patient population, including older adults and those with multiple comorbidities, ensures the generalizability of the findings. However, the unequal distribution of comorbidities, with a higher proportion of advanced-age and comorbid patients in the HFRT group, may introduce confounding variables. The absence of chemotherapy sensitizers limits the study’s ability to assess the synergistic effects of hypofractionation with systemic treatments. Furthermore, a shorter follow-up period restricts the assessment of long-term efficacy and late toxicities, while the lack of severe (grade 4) toxicities leaves uncertainties about rare but severe side effects. Finally, the similar response rates between the two treatment arms may suggest that the benefits of hypofractionation may be modest, warranting further investigation with larger sample sizes and more sensitive outcome measures.

## Conclusion

This study demonstrates that 42.72 Gy in 16 fractions, particularly when combined with brachytherapy, offers a promising treatment option for cervical cancer, while HFRT offers comparable clinical outcomes and toxicity profile to CFRT. The reduced treatment duration and associated cost savings can improve patient convenience and alleviate strain on healthcare resources, making HFRT a potentially transformative option for cervical cancer treatment, especially in low-resource countries like those in Africa. The study emphasizes the advantages of HFRT in improving treatment accessibility and reducing resource demands while also underscoring the importance of closely monitoring its toxicity profile in comparison to CFRT.

## Data Availability

The raw data supporting the conclusions of this article will be made available by the authors, without undue reservation.
